# Progress in Surgery

**Published:** 1899-08-26

**Authors:** 


### Progress in Surgery.

### SURGERY OF THE INTESTINES.

Comparatively few cases of ruptured intestine are recorded of the many which occur. A typical example is given by Dr. A. Sheen.[1] In this case a man received a blow in the abdomen from a knee, and on admission appeared to be dying. At the post-mortem a linear tear was found at the junction of duodenum and jejunum. Rupture of the intestine is not necessarily fatal if operation be early performed, as in the following case under the care of Mr. Walsham.[2] In this case there was no external wound, but the operation was performed within two and a half hours of the accident. It may be mentioned that the liver dulness persisted. The patient was a boy, aged 13, who had been run over by a post-office van. The intestine was sutured, and the boy recovered.

The treatment of intestinal obstruction has much improved of late years, and when patients die after operation it is usually due to delay and to some lesion of a fatal character *per se*. For instance, there is frequently inflammation, ulceration, and sometimes perforation of the gut above a stricture, as has been shown by Kocher. The ulceration is not due to pressure of hard fæcal masses, for the contents are commonly liquid. It is probable that ecchymoses occur in the mucous membrane of the congested and inflamed bowel, which ,so alter the nutrition that micro-organisms can wander in and set up local and start general septic processes. Hence it is of the greatest importance to relieve the over-distended bowel and remove the septic contents, and in many cases this is more important than the immediate removal of the cause. Indeed, patients sometimes die purely from the toxæmia, although the cause of the obstruction be successfully removed. The late Greig Smith drew attention to the advantages of incisions into the bowel, and the practice is followed by other surgeons with the best results. Thus Mr. Ernest Maylard says he now always pursues this course with the best results.[3] In fact he frequently empties the gut before seeking for the cause of the obstruction, making an evacuatory incision and passing the fingers along the bowel to evacuate the contents. Maylard suggests the same measure for the paralytic distension of peritonitis, and claims success in its employment.

An interesting case of double obstruction by band is recorded by Dr. W. J. Taylor, the second obstruction occurring about two years after the first. In both instances operation was successful.[4]

Fabre has only been able to collect nine cases of intestinal obstruction from torsion of the mesentery. It is usually due to inflammatory bands or congenital mesenteric fossæ. In partial torsion a small coil of intestine is shut off, but in total torsion the whole jejunum and ileum may be isolated and changed to a closed cavity. The prognosis is almost hopeless, and the diagnosis almost impossible before operation. Enterotomy is useless, and the only hope is in untwisting the mesentery.[5]

Volvulus is a deadly affection, difficult to treat successfully. Littlewood has had the good fortune to meet with eight cases in three years, and save three of the patients. Four were cases involving the large intestine and three involved the small intestine. The urgency of the condition renders adequate knowledge of its treatment imperative. Hence a brief *résumé* of some of the cases may be instructive. Case I. Volvulus of large intestine, including the whole descending colon; great pain and rapidly increasing distension; general peritonitis found; volvulus untwisted and incised. Death. Case II. Volvulus of cæcum and contiguous large and small intestine, the cæcum forming the apex of the volvulus. Recovery. Case III. Volvulus of sigmoid, untwisted, incised, about a quart of fæcal fluid coming away. Case IV. Volvulus of sigmoid, peritonitis. Recovery. Case V. Gangrenous volvulus of small intestine, enterectomy by suture, recovery after giving way of part of the volvulus. A slough about a foot long—including the whole volvulus—came away, followed by fistulous openings and attacks of partial obstruction. A secondary resection of gut was performed.. Case VI. Volvulus of small intestine with an ulcer of the mesentery at point of twisting. Volvulus drained, reduced. Death. Case VII. Volvulus of small intestine with tuberculous glands in its mesentery. Death.

Mr. Littlewood remarks how seldom it is possible to make a correct diagnosis. Of the seven cases he only correctly diagnosed one.

It is difficult to follow recent methods of anastomosis of the intestine, they are so numerous, and many operators still prefer simple suture to the use of the various mechanical contrivances. Alexander MacLennan gives a good *résumé* of the principal methods of anastomosis.[6] He gives an ingenious method. In this it is essential to distinguish the proximal from the distal extremity of the gut; indeed, life may depend on this. A decalcified bone tube is introduced into the proximal end of the gut and ligatured at the level of one of the grooves. By an ingenious device with silkworm gut the distal end is invaginated, and then the tube-lined proximal end passed into it, and the distal end fixed in the second groove, where it is ligatured, the ligature cut short, and its perforation closed by a purse-string suture.

Dr. Ernest Laplace has invented handled rings for holding the intestines together whilst suturing them.[7] The forceps, being separable into two halves, can be gently withdrawn from a small aperture still unsutured. and the anastomosis is completed by adding one or two sutures. The method has been much praised by many American surgeons, and the principle is regarded as that of the future.

A most interesting pathological curiosity has been described by Dr. Chas. K. Briddon. An infant for some time defæcated naturally, but soon began to defæcate through the umbilicus instead. This was followed by prolapse of the ileum through the unclosed omphalo-mesenteric duct.

Emphysema from malignant disease of the sigmoid is not a common event. Such a case is recorded by Dr. H. Kerr, in which the emphysema extended over the back and chest before the patient died.[8]

An interesting case of prevesical hernia was described by Mr. S. H. Makins before the Royal Medico-Chirurgical Society, in which the gut had undergone subacute strangulation. It appeared that there was a diverticulum of the peritoneum in that situation.[9]

[1] Brit. Med. Jour., Feb. 25, 1899. [2] Lancet, Jan. 7, 1899. [3] Brit. Med. Jour., April 8, 1899. [4] Annals of Surgery, Feb. 7, 1899. [5] Epit. Brit. Med. Jour., Mar. 25, 1899. [6] Lancet, Feb. 25th, 1899. [7] Annals of Surgery, Mar., 1899. [8] Lancet, Nov. 26, 1898. [9] Brit. Med. Jour., April 15, 1899.

